# MECs: "Building Blocks" for Creating Biological and Chemical Instruments

**DOI:** 10.1371/journal.pone.0158706

**Published:** 2016-07-20

**Authors:** Douglas A. Hill, Lindsey E. Anderson, Casey J. Hill, Afshin Mostaghim, Victor G. J. Rodgers, William H. Grover

**Affiliations:** Department of Bioengineering, University of California Riverside, Riverside, CA, United States of America; University of Illinois at Chicago, UNITED STATES

## Abstract

The development of new biological and chemical instruments for research and diagnostic applications is often slowed by the cost, specialization, and custom nature of these instruments. New instruments are built from components that are drawn from a host of different disciplines and not designed to integrate together, and once built, an instrument typically performs a limited number of tasks and cannot be easily adapted for new applications. Consequently, the process of inventing new instruments is very inefficient, especially for researchers or clinicians in resource-limited settings. To improve this situation, we propose that a family of *standardized multidisciplinary components* is needed, a set of “building blocks” that perform a wide array of different tasks and are designed to integrate together. Using these components, scientists, engineers, and clinicians would be able to build custom instruments for their own unique needs quickly and easily. In this work we present the foundation of this set of components, a system we call Multifluidic Evolutionary Components (MECs). “Multifluidic” conveys the wide range of fluid volumes MECs operate upon (from nanoliters to milliliters and beyond); “multi” also reflects the multiple disciplines supported by the system (not only fluidics but also electronics, optics, and mechanics). “Evolutionary” refers to the design principles that enable the library of MEC parts to easily grow and adapt to new applications. Each MEC “building block” performs a fundamental function that is commonly found in biological or chemical instruments, functions like valving, pumping, mixing, controlling, and sensing. Each MEC also has a unique symbol linked to a physical definition, which enables instruments to be designed rapidly and efficiently using schematics. As a proof-of-concept, we use MECs to build a variety of instruments, including a fluidic routing and mixing system capable of manipulating fluid volumes over five orders of magnitude, an acid-base titration instrument suitable for use in schools, and a bioreactor suitable for maintaining and analyzing cell cultures in research and diagnostic applications. These are the first of many instruments that can be built by researchers, clinicians, and students using the MEC system.

## Introduction

The importance of instrumentation in research, industry, and healthcare is difficult to overstate. From a simple incubator in a biology lab to a sophisticated genetic analyzer in a hospital, instruments provide essential automation and quantification. Consequently, new instruments accelerate research and open doors to potentially lifesaving diagnostics and treatments.

New instruments that are purely electronic can be designed and built relatively easily because they are comprised of electronic components (resistors, capacitors, integrated circuits, etc.) that were designed for easy interconnectivity. However, many instruments (especially those in the chemical, biological, and medical fields) are not only electronic but also include fluidic, mechanical, and optical elements. With no standardized components designed for interconnectivity, new instruments that combine fluidic, mechanical, optical, and electrical components are relatively slow and inefficient to build. As a result, scientists and clinicians may recognize the need for a new instrument in their work but be unable to obtain an instrument or build their own because of the specialized training, equipment, and time needed to design and build new instruments.

In this work, we introduce “Multifluidic Evolutionary Components” (MECs), a system of “building blocks” that can be used to create complete, functional instruments quickly and easily. The term “multifluidic” recognizes the fact that real-world chemical and biological instruments are often *multiscale*, operating on fluid volumes ranging from nanoliters to milliliters and beyond. While microfluidic “building blocks” have been demonstrated that allow for custom microfluidic instruments [1–4], these systems are purely microfluidic and cannot also operate on the milliliter-and-larger volumes commonly encountered in real-world chemical, biological, and medical instruments. Our term “multifluidic” also reflects the fact that real-world biological and chemical instruments are often *multidisciplinary*, containing not only fluidic but also mechanical, optical, and electronic parts. Most existing microfluidic “building blocks” are purely fluidic and do not provide mechanical, optical, or electronic components. Each MEC “building block” performs a fundamental function that is commonly found in biological or chemical instruments, functions like valving, pumping, mixing, controlling, and sensing. By combining MECs together, researchers can build custom biological or chemical instruments quickly and easily.

**Fig 1:**
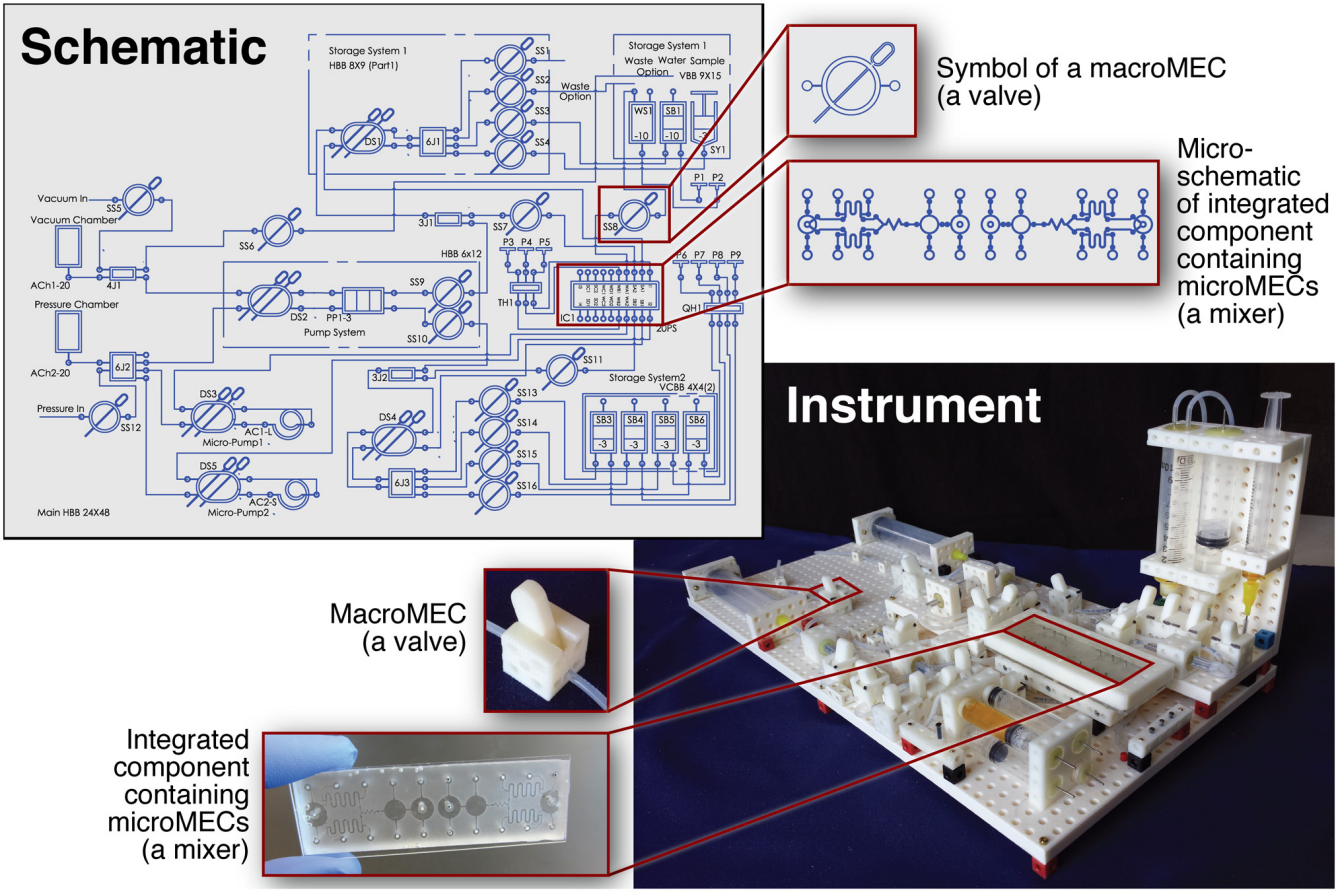
Overview of designing and building a custom instrument using the Multifluidic Evolutionary Component (MEC) system. The instrument is designed by arranging MEC symbols on a schematic, then the schematic is used to guide the assembly of MEC “building blocks” into the finished instrument. For many applications involving manipulating fluids on the milliliter scale, the “off the shelf” macroMEC components (like the manual valve shown) are adequate for building complete instruments. For instruments that must manipulate fluids on the microliter or nanoliter scale, microMECs can be arranged into a micro-schematic (like the mixer chip shown), fabricated using conventional microfabrication techniques, packaged in a MEC shell, and plugged into the rest of the instrument.

Fig 1 provides an overview of the process used to create an instrument using MECs (in this case, a fluidic router capable of pumping and mixing various fluids). Instrument design begins with a user arranging MEC symbols into schematics. Each symbol is linked to a physical definition of the component. Two types of MECs are used: *macroMECs* (milliliter-scale off-the-shelf components which can be “snapped together” for many applications) and *microMECs* (nanoliter- or microliter-scale components which are combined in a micro-schematic and made using any conventional microfluidic technique like etching, embossing, or soft lithography). After the design of the instrument is finalized, the schematic guides the user in assembling macroMECs and microMECs into a complete and functional instrument.

## Methods

### “Evolutionary” design principles

The MEC system was designed to be “evolutionary,” to easily adapt to a wide range of new applications and easily accommodate new components. To accomplish this, we incorporated design principles from two existing systems that are already “evolutionary” by our definition: *biological cells* and *electronic components*. The specific qualities of cells and electronics that we attempted to replicate in the MEC system include:

#### Packaging

**Fig 2:**
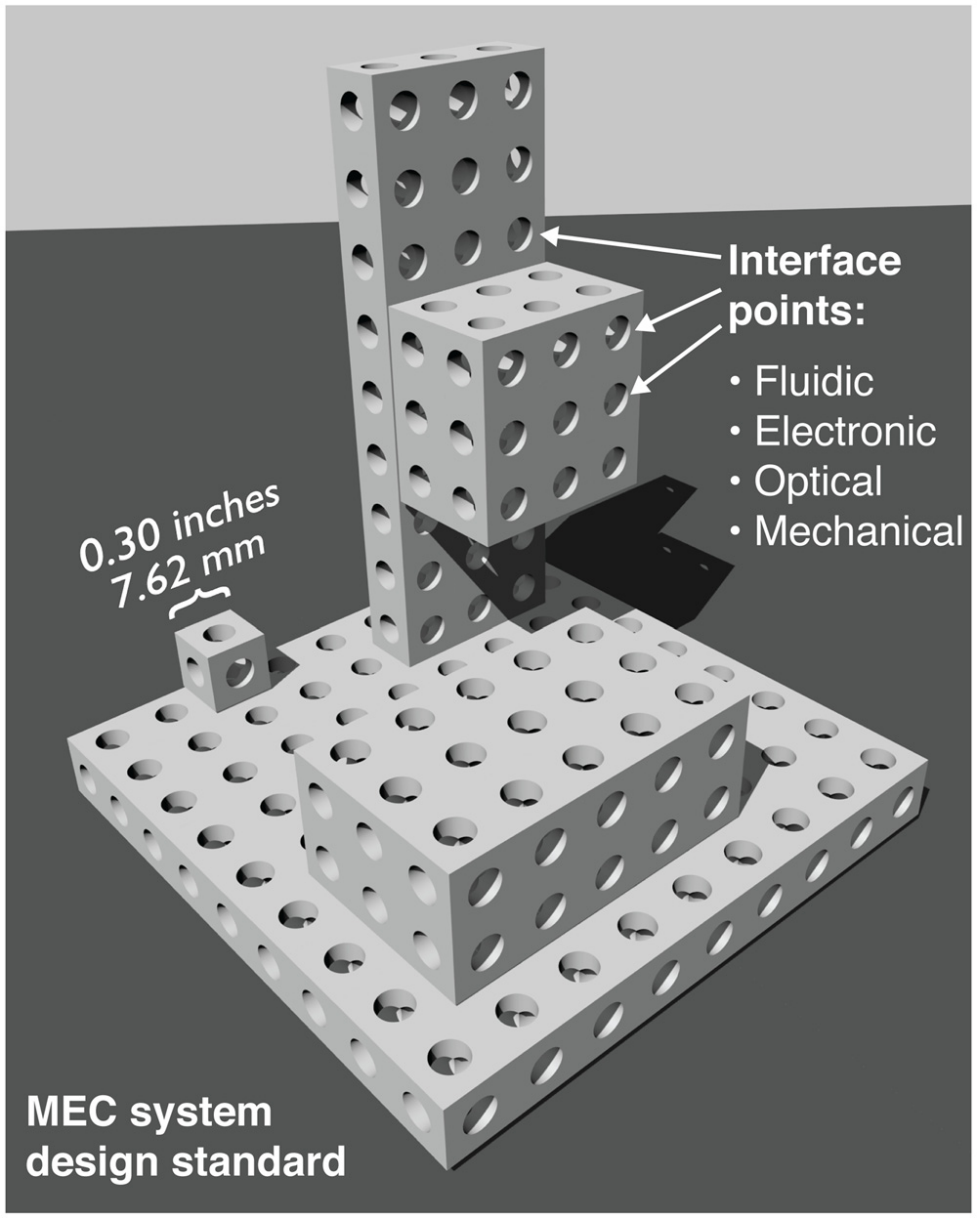
Design standard for the MEC system. Standardized sizing and spacing allows MECs to be connected together in all three spatial dimensions, and interface points (circles) can be designed to allow fluid, electricity, or light to flow between MECs.

Biological cells are packaged in a cell membrane that controls mass transport into and out of the cell, and electronic components like integrated circuits are typically packaged in cases with metal pins for input/output connectivity. In both cases, the package’s contents and function may vary widely in different cell or chip types, but the package itself remains relatively constant. By wrapping contents in standardized packages, components can be interconnected easily and efficiently. Similarly, in the MEC system a structured package design (Fig 2) enables a variety of different fluidic, electronic, optical, and mechanical components to be interconnected with each other.

#### Symbolic design

DNA provides a set of symbols that define the design and function of a cell, and schematics serve the same purpose in electronics. Likewise, in the MEC system design begins at the symbolic level, where each component has a symbol linked to a physical definition. Several MEC symbols are combined to generate a schematic, and the final instrument is assembled by following the schematic like a set of instructions. Schematics facilitate the design of complex MEC-based instruments, enable multiple designers to easily communicate and collaborate with each other, encourage the sharing of MEC instrument designs, and allow new MEC instruments to build on the designs of older MEC instruments.

#### Interdisciplinary components

The functions of biological cells span many different disciplines, including optics (rod and cone cells in the eye), electronics (nerve cells), mechanics (bone cells), and so on. Similarly, many electronic components are also optical (LEDs and photodiodes), mechanical (solenoids and strain gauges), and so on. Integrating multiple fields or disciplines into a set of components greatly expands the range of applications for those components. For this reason, the MEC system includes components with fluidic, electronic, mechanical, and optical functionalities, packaged in a way that facilitates interconnection.

#### Standardized three-dimensional interfaces

Biological cells can communicate and exchange matter with their neighbors in all three spatial dimensions. Interestingly, electronic circuits are largely planar and communicate mainly in two dimensions, a limitation that electrical engineers have struggled with for the last half century [5]. To avoid the limitations of two-dimensional interconnectivity, the MEC system uses a three-dimensional interface standard shown in Fig 2. Consistently-sized and regularly-spaced interface points (the circles in Fig 2) can contain mechanical, electrical, fluidic, or optical interfaces. Fluid, light, or electric current flow from one MEC to another through these interfaces.

### Open standard for MEC interfaces

To make it easier for other researchers to develop their own components that can interface with the MEC system, we are maintaining an open standard for MEC interfaces. The current version of the standard is summarized below, and the latest version is available for reference on our website [6].

**Mechanical interface:** As depicted in Fig 2, MEC blocks are designed in 0.3 inch increments (a block’s nominal size in each dimension can be 0.3 inches, 0.6 inches, 0.9 inches, and so on). Each dimension of a MEC is actually designed to be 0.005 inches smaller than a 0.3-inch multiple to allow space for two MECs to be connected side-by-side (for example, the 0.3 inch thick mechanical baseboard MEC is actually 0.295 inches thick). Surfaces of MECs contain rectilinear arrays of holes spaced on 0.3 inch increments. The diameter of each hole changes along the depth of the hole: the topmost (and bottommost) 0.0865 inches of the hole has a diameter of 0.125 inches, and the middle of the hole has a diameter of 0.092 inches. The larger 0.125-inch diameter portion of the hole is designed to receive a 0.125 inch diameter and 0.175 inch long pin. Using these pins, two MECs can be connected together using a simple and reversible friction-fit assembly. Alternately, a standard #2–56 screw can fit through the 0.092-inch-diameter portion of a hole and self-thread into a 0.076-inch-diameter hole on a MEC part. This provides a mechanically stronger alternative to the pins for connecting two MECs together.

**Fluidic interface:** The holes on 0.3-inch-spaced increments can be sized to accommodate flexible tubing (e.g., Tygon brand) with 3/32 inch outer diameter and a 1/32 inch inner diameter. A short length of tubing inserted into a hole can then serve as a fluidic socket. A 20-gauge rigid tube (e.g., stainless steel) can then be inserted into the tubing-based fluidic socket. This creates a simple and reversible friction-fit fluidic connection between two MECs. Alternately, two MECs whose faces do not touch (and therefore cannot be easily connected by the rigid tube) may be connected using a length of flexible tubing. In this case, a 20-gauge rigid tube is permanently inserted into each MEC (extending at least 0.29 inches out of the MEC surface), and flexible tubing is slipped onto these tubes to form a reversible fluidic connection between the two MECs. For applications that must withstand higher fluid pressures, a retaining ring (0.125 inches outer diameter, 0.086 inches inner diameter, and 0.175 inches long) can be slipped on the outside of the flexible tubing at each MEC to serve as a compression fitting.

**Electrical interface:** The 0.3-inch-spaced holes described above can also accommodate metal pins, conductive rubber pins, metal springs, or metal screws to provide electrical connectivity between MECs.

**Optical interface:** Optical elements like light emitting diodes, phototransistors, and fiber optics can be designed into the 0.3-inch-spaced holes and used to provide optical interfaces between MECs.

### Fabrication of MECs

As long as MECs are designed and packaged to interface together, virtually any material or fabrication technique can be employed to make MECs. In this proof-of-concept demonstration, we mostly used rapid prototyping (3D printing) and CNC machining to fabricate the larger-scale macroMECs. Two 3D printers were used: a fused desposition modeling (FDM) printer (Dimension Elite by Stratasys, Eden Prairie, MN) which uses melted acrylonitrile butadiene styrene (ABS) to form a part, and a stereolithography (SLA) printer (Form 1+ by Formlabs, Cambridge, MA) which uses ultraviolet light to cure liquid methacrylate resins into a solid part. To fabricate microfluidic integrated components that contain microMECs, we used a custom casting process we developed based on soft lithography [7] for polydimethylsiloxane (PDMS) components, and conventional photolithography and wet etching [8] for borosilicate glass components.

### MacroMECs

**Fig 3:**
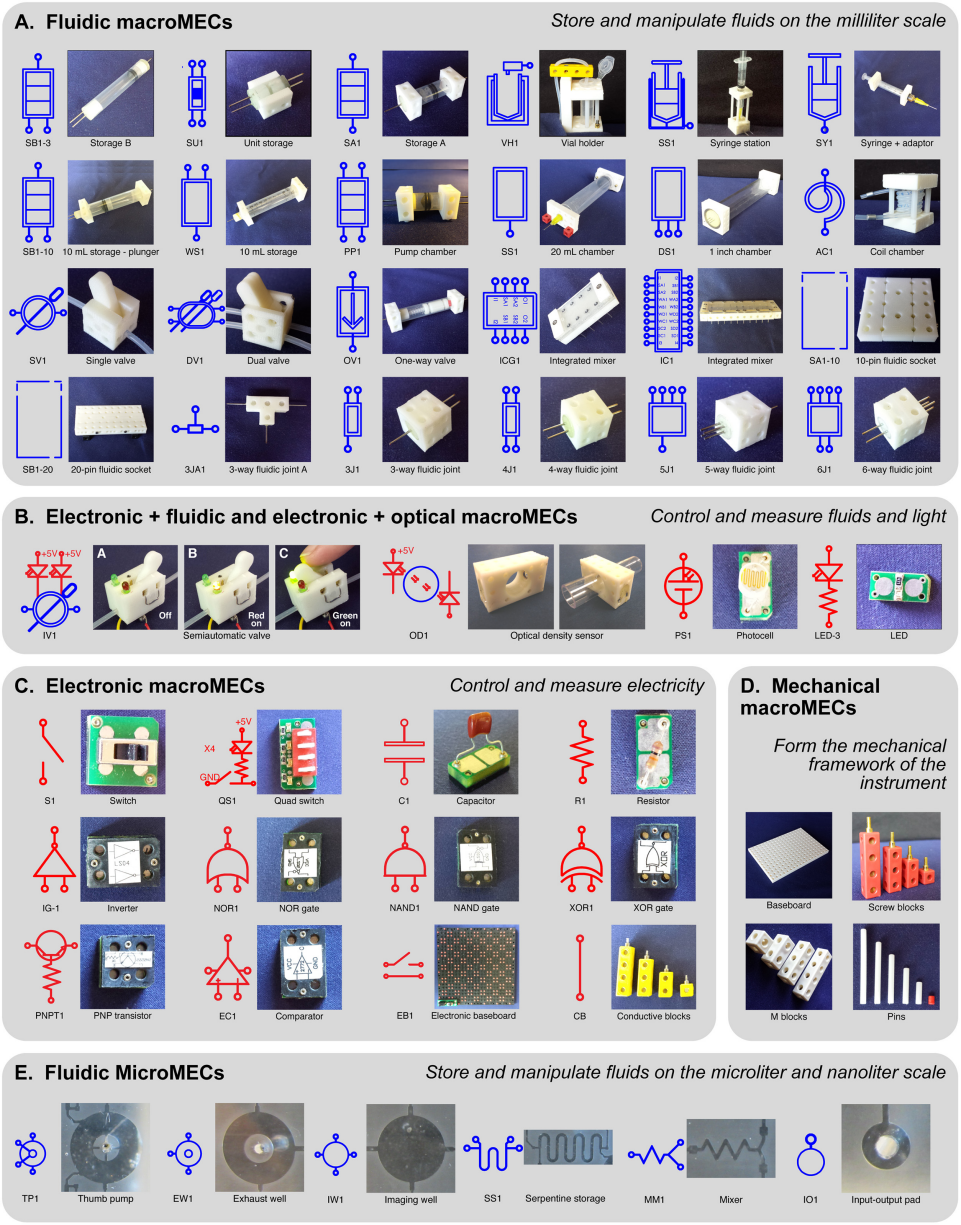
Selected Multifluidic Evolutionary Components (MECs), including macroMECs that can be “clicked together” (A–D) and microMECs that are fabricated into microfluidic integrated components (E).

Each of the macro-scale Multifluidic Evolutionary Components (macroMECs) shown in Fig 3 performs a fundamental function that is commonly found in real-world instruments. They include fluidic macroMECs for storing, pumping, and controlling milliliter-scale volumes of fluids (Fig 3A), electronic/fluidic and electronic/optical macroMECs that control and measure fluids and light (Fig 3B), electronic macroMECs that perform logical and sensing operations (Fig 3C), and mechanical macroMECs that form the mechanical framework or skeleton for MEC-based instruments (Fig 3D).

#### Fluid storage

A variety of fluid storage MECs contain fluid samples, reagents, and waste in MEC-based instruments. Some of these MECs (like SB1-3 in Fig 3A) are modeled after syringes and have moveable plungers that separate the fluidic and pneumatic sides of the component. Air pressure or vacuum applied to the pneumatic side empties or fills the fluidic side of the storage MEC, thereby creating a storage reservoir that doubles as a simple pump. In addition, a family of unit storage MECs (like SU1 in Fig 3A) can contain variable volumes, with the precise volume determined by the length of an internal spacing pin. Other storage MECs interface to more conventional fluid reservoirs like microcentrifuge tubes (VH1 in Fig 3A), syringes (SS1), and milliliter-scale containers (SB1-10 and WS1).

#### Vacuum and pressure chambers

Portability is important for many instruments intended for point-of-care or field applications. Portable MEC-based instruments are powered by an onboard pneumatic vacuum and pressure chamber MECs (DS1 in Fig 3A). These chamber MECs are analogous to batteries in electronics; they provide a pressure differential to pull or push fluid through other components.

#### Manual valves

Valve macroMECs control the flow of fluid in a MEC-based instrument. As the operator toggles the lever on the mechanical pinch valve MEC (SV1 in Fig 3A), the lower part of the lever either pinches the internal tube closed (stopping the flow of fluid through the valve) or releases the tube (allowing fluid flow to resume). A dual pinch valve MEC (DV1 in Fig 3A) has four fluid connections and three states. In the first state (switch up), fluid flows between connections 1 and 2. In the second state (switch down), fluid flows between connections 3 and 4. In the third state (switch in the middle), all fluid flow is stopped. This behavior (called “break before make” in electronic switches) is particularly useful in applications requiring switching between two sources (say, pneumatic pressure and vacuum) that should never be connected together during valve operation.

#### Pumps

The ability to pump fluid is a fundamental requirement in many biological and chemical instruments. The MEC system provides several different pumping methods. First, as described above, some fluid storage MECs (like SB1-3 in Fig 3A) are equipped with internal plungers and function as simple pumps; they are well-suited for operations such as rinsing or waste removal that require the entire contents of a storage MEC to be delivered. Second, by combining a pump chamber MEC (PP1 in Fig 3A) with a dual valve (DV1) and two single valves (SV1), a milliliter-scale “unit volume” pump is created that is useful for pumping known volumes of fluids between MECs. The volume dispensed from the pump with each actuation is determined by the volume of the pump chamber. Third, a coil chamber MEC (AC1 in Fig 3A) combined with a dual valve (DV1) creates a microliter-scale unit volume pump. The volume of air pumped per actuation ranges from a few to hundreds of microliters and is determined by the volume of compressed air held in the coil chamber MEC.

#### Fluidic sockets

Integrated components (microfluidic chips containing microMECs, described below) connect to the rest of the MEC system through fluidic socket macroMECs (SA1-10 or SB1-20 in Fig 3A). These sockets provide an array of holes containing elastic tubing that receives stainless steel fluidic pins on the integrated components. Fluid passes between the microfluidic integrated component and the rest of the MEC-based instrument through these sockets.

#### Semiautomatic valves

Complex instruments for biological and chemical applications can require a large number of valves. If these valves are actuated by hand, like the mechanical pinch valve MECs, actuation steps may be missed or improperly timed. To reduce this risk while still keeping the overall component cost low, the “semiautomatic” valve (IV1 in in Fig 3B) combines computer control with manual actuation. This valve contains LEDs that interface to an Arduino-based microprocessor MEC. The red light indicates to the operator that a particular valve needs to be toggled. Once the valve is switched, the red light turns off and the green light comes on, indicating to the operator that the valve was properly switched. The green light signal can also be sent back to the microcontroller to confirm that the valve’s state is now correct; the microcontroller could then turn off the light. This combination of manual operation with computer-guided control reduces component cost while still supporting complex operations and reducing error rates.

#### Electronic MECs

A variety of electronic macroMECs support electrical and logical functions in MEC-based instruments (Fig 3C). Discrete components like resistors (R1) and capacitors (C1) plug into a two-pin macroMEC adaptor. This allows a large array of standard electronic components to be packaged as macroMECs and incorporated into MEC-based instruments. Switches (S1 and QS1) and transistor (PNPT1) MECs control the flow of electric current between two or more MECs. A family of logic macroMECS (IG-1, NOR1, NAND1, and XOR1) can perform all the standard Boolean logic functions. Using logic MECs, the operation of an instrument can be defined and controlled. Finally, the the power and ground baseboard (EB1 in Fig 3C) contains an embedded printed circuit board. This baseboard contains an array of power and ground connections arranged in a checkerboard pattern, and a jack for connecting a standard 5 V wall-mounted power supply. This baseboard provides many convenient locations for connecting and powering electronic macroMECs.

#### Sensor and measurement MECs

A variety of sensors can be connected to a macroMEC adaptor and used in the MEC system; for example, a photocell MEC (PS1 in Fig 3C) enables MEC-based instruments to measure light intensity. A more specialized sensing MEC, the optical density MEC (OD1 in Fig 3B), includes a light source (an LED) and light sensor (a photodiode) oriented directly across from each other on a transparent tube. By measuring how much light from the LED is detected at the photodiode, the optical density MEC can measure many different properties of the fluid flowing through the tube, including solute concentration (using the Beer-Lambert law), turbidity or optical density (a measure of how many particles or cells are in the fluid), fluid level sensing (using the different indices of refraction for gas and liquid), and so on.

#### Mechanical MECs

A set of structural macroMECs (Fig 3D) provides the mechanical framework for MEC-based instruments. Hollow blocks can contain plastic pins (for connecting MECs together mechanically), metal rods (for electrical MEC connections), tubing (for fluidic MEC connections), etc. The mechanical baseboard MECs provide a foundation for MEC assembly and can be oriented horizontally or vertically (see Fig 1 for examples of both orientations).

### MicroMECs

Like the macroMECs described above, micro-scale Multifluidic Evolutionary Components (microMECs) perform fundamental functions like pumping, mixing, and storage, only on a smaller scale (microliters and nanoliters). Each of the microMECs has a symbol associated with it (Fig 3E). A few of the microMECs are detailed below.

#### Micropumps

The thumb pump microMEC (TP1 in Fig 3E) contains a microliter-scale volume of liquid or gas. When pressed by a finger in a manner described previously [9], the pump is designed to push fluid downstream to other microMECs. An exhaust well microMEC (EW1 in Fig 3E) vents displaced liquid or gas from the chip.

#### Fluid storage and metering

The serpentine storage microMEC (SS1 in Fig 3E) holds a fixed fluid volume of 400 nL. It is designed with two sets of inlets and outlets. Other nanoliter- or microliter-scale storage microMECs can be designed using differently-sized channels. Additionally, the imaging well microMEC (IW1 in Fig 3E) has a transparent glass top for imaging its contents.

#### Mixing

A simple mixing microMEC (MM1 in Fig 3E) is designed to combine the contents of two channels.

#### Fluidic input/output

The circular input/output pad (IO1 in Fig 3E) provides a fluid interface between other microMECs in an integrated component and a fluidic pin (a stainless steel tube). This pin then serves as the fluidic interface to macroMECs as described above.

### Integrated components

**Fig 4:**
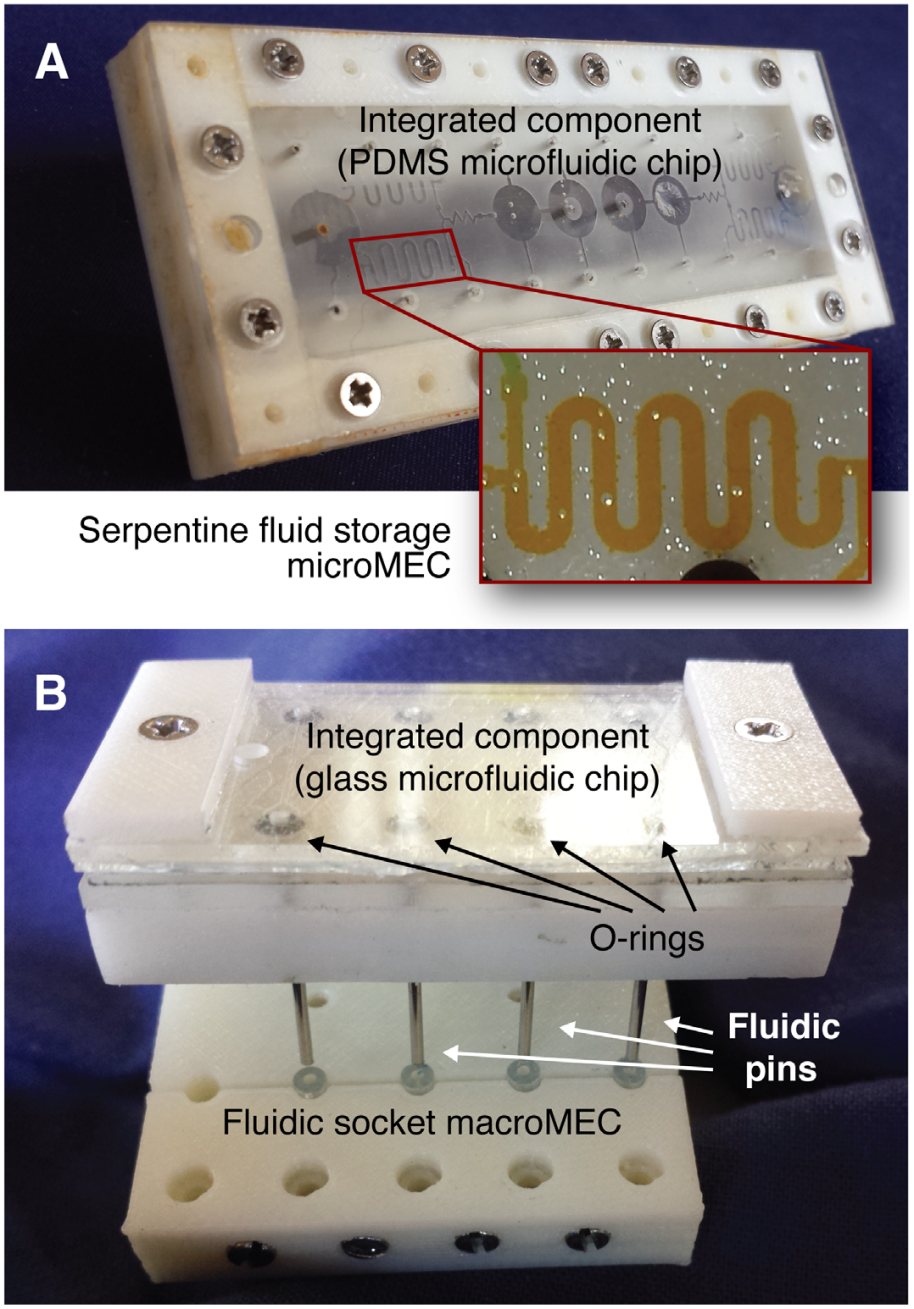
Two *integrated components*, microfluidic chips containing several microMECs combined together and fabricated using soft lithography of PDMS (A) and wet etching of glass (B). The integrated components are packaged in a MEC-standard shell that plugs into the fluidic socket macroMECs (SA1-10 or SB1-20 in Fig 3A). By packaging a microfluidic chip in a MEC shell in this manner, virtually any microfluidic chip can be integrated into the MEC system.

MicroMECs are combined into *integrated components*, chips that can be fabricated using conventional microfabrication techniques, packaged inside a MEC-standard mechanical shell, and plugged into the fluidic socket macroMEC described above. Fig 4 shows two examples of integrated components, one fabricated in PDMS using soft lithography (Fig 4A) and one fabricated in glass using wet chemical etching (Fig 4B).

## Results

To evaluate the performance of the Multifluidic Evolutionary Component system, we used MECs to build several test instruments. Here we present three MEC-based chemical and biological instruments: a fluidic router capable of sending fluids between several storage MECs, a prototype acid-base titration instrument for educational use, and a research-grade bioreactor capable of measuring the growth rate of cells in culture.

### Fluidic router

**Fig 5:**
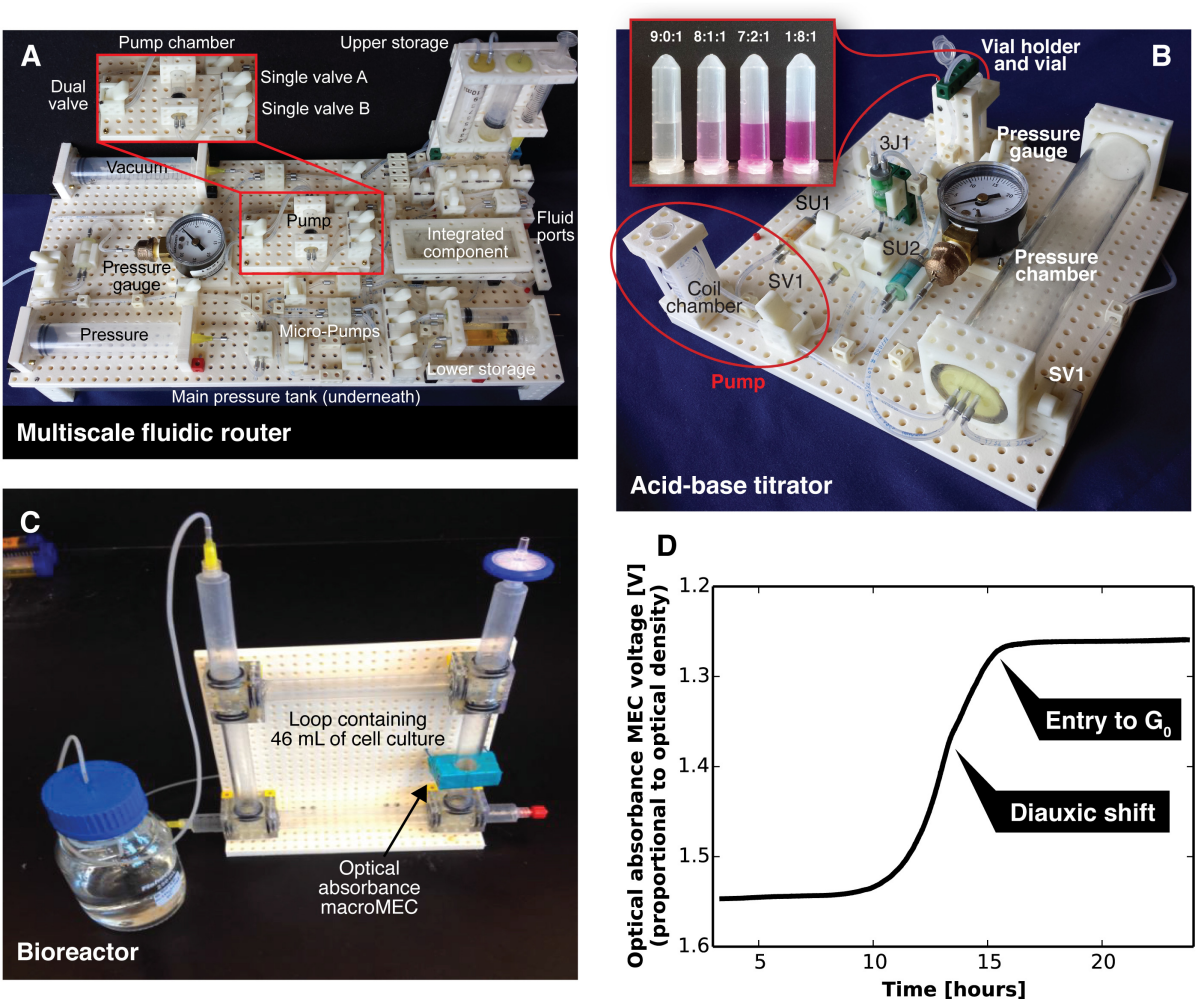
Three sample instruments built using the MEC system. (A) A MEC-based multiscale fluidic router designed to mix fluids from nine different fluid storage MECs in volumes spanning five orders of magnitude (from 400 nanoliters to 10 milliliters). (B) A MEC-based acid-base titration instrument suitable for educational applications in classrooms. By toggling the manual valves SU1, SU2, and SV1, different ratios of water: sodium hydroxide solution: phenolphthalein are delivered to a microcentrifuge vial. The observed range of phenolphthalein colors (from clear in pure water to pink in 4.0 × 10^−5^ mass concentration NaOH) confirms the successful operation of the instrument. (C) A MEC-based bioreactor capable of culturing cells. The optical density macroMEC (OD1 in Fig 3B) measures the concentration (via optical density) of the cells as they grow and react to stimuli. (D) A growth curve obtained by using the bioreactor in C to culture *Saccharomyces cerevisiae* yeast cells. The bioreactor is sensitive enough to detect important metabolic checkpoints in the growing yeast, including the diauxic shift (when the yeast cells switch from glycolysis to the aerobic oxidation of ethanol) and the entry into G_0_ (when the yeast cells exhaust all nutrients and enter stationary phase).

To demonstrate the complexity of instruments that can be built using MECs and the multiscale capability of the MEC system, we developed the fluidic router shown in Fig 5A. It was built from our current set of standard MEC components and combined both macroMECs and microMECs in the same instrument. The instrument is designed to route fluids between nine fluid storage MECs in several different ways, in volumes spanning six orders of magnitude (from nanoliters to milliliters). The fluidic router also demonstrates a serial fluidic bus design in which several fluid storage MECs are connected in series by a single channel. This design accommodates additional storage MECs with ease and reduces the risk of cross-contamination by supporting a rinse operation between fluid transfer operations. During bus rinsing, the contents of the serial bus channel are flushed to a waste storage MEC to remove residues from the previous fluidic operation and prepare for the next operation. A larger storage MEC (WS1 in Fig 3) was added to the MEC system to provide rinse fluid for this purpose. The router instrument also demonstrates the ability to interface between microMECs and macroMECs using the fluidic socket.

During operation, the router instrument’s pressure tanks are first pressurized as described earlier. The pressure tanks shown on the top of the fluid router were selected from our original standard component set (SS1 in Fig 3A). Unfortunately, these tanks provided only enough air for a few pump actuations, so a larger air chamber (DS1 in Fig 3A) was developed and was mounted on the bottom of the fluidic router in Fig 5A. This new air chamber became our standard, and was fabricated in different lengths for different air capacities. The user loads a sample into the instrument via a syringe. The central pump can be used to transfer fluid to the lower reservoirs, or fluid can be directly loaded from the side fluid ports on the right in Fig 5A. To transfer fluid using the central pump, first single valve A and two other valves are opened to allow passage of fluid from the syringe or storage MEC to the pump chamber. The dual valve on the pump is toggled to allow vacuum to be applied to the pump chamber, pulling back the plunger. As the pump plunger pulls back, it draws fluid from the storage MECs into the pump chamber, with the precise volume determined by the size of the chamber. Single valve A is then turned off. One of the four lower storage MECs is then selected by toggling the appropriate single valve. Single valve B is then turned on. By switching the dual valve on the pump, pneumatic pressure is applied to the plunger in the pump chamber, pushing the plunger forward and forcing fluid out of the pump chamber into the destination lower storage MEC. In this manner, fluid can be moved between any of the storage MECs in either direction.

To mix fluids on the milliliter scale, the pump is used to route fluid from two or more storage MECs into a third storage MEC. To mix fluids on the microliter to nanoliter scale, the dual valve is switched to divert fluids through the serpentine storage microMECs on the microfabricated integrated component. Once the 400 nL serpentine storage microMECs are loaded, the coil pump microMEC is used to drive the fluid out of the serpentine storage microMECs, through the mixer, and into the imaging well. The coil pump is made from the standard dual valve and a small length of tubing. It is noteworthy that this pumping application was not envisioned when the dual valve was developed; this is an example of how standard MECs can be used in novel and unexpected ways. Fig 5A shows a milliliter-scale volume of fluid (dyed yellow) that has been delivered from a syringe (or one of the other storage units) to one of the lower storage MECs by the instrument, and Fig 4A shows a serpentine fluid storage microMEC filled with 400 nL of fluid. The PDMS integrated component shown in the fluidic router was fabricated using an experimental casting process that caused some bonding problems and leakage between the PDMS and glass surfaces, but the socketing and interconnection of the integrated component still functioned as intended.

### MEC-based titration instrument

To demonstrate the potential of the MEC system in educational applications, we used our standard MEC components to construct the acid-base titration instrument shown in Fig 5B. This system mechanizes the steps involved in performing an acid-base titration, a classic chemistry lab experiment used to measure the equilibrium constant of a weak acid or base. The MEC-based titration instrument in Fig 5B delivers unit volumes (100 *μ*L) of either water, sodium hydroxide solution (5.0 × 10^−5^ mass concentration), or phenolphthalein solution (0.1% in 50% ethanol) to a vial. The titration instrument also utilizes the new pressure chamber macroMEC described above. This pressure chamber makes the MEC-based titration instrument portable and suitable for use in the field, in classrooms, or at the point of care. The reuse of this new pressure chamber MEC in multiple instruments demonstrates the natural evolution of MEC components: the original pressure chamber MEC had less utility then the new pressure chamber MEC, so the original MEC was naturally replaced.

During operation, a syringe is used to load pressurized air into the pressure chamber (DS1) until the chamber’s contents reach the desired pressure (measured by the gauge in Fig 5B). This gauge MEC is an example of how an standard industry component like a gauge can be adapted to the MEC system (in this case, by attaching the gauge to a MEC mechanical baseboard and fitting the gauge with a MEC standard tubing interface). Fluid is then loaded by syringe into each of the unit chambers (SU1 and SU2). The unit chambers can be loaded one at a time or in parallel. Next, the dual valve (SV1) is used to load the coil chamber (AC1) with pressurized air. The volume of pressurized air in the pump chamber is determined by the length of tubing in the coil. The dual valve is then switched back to apply pressure from the coil chamber to the unit chambers, forcing their fluid contents into the vial. With each valve actuation, a unit volume from one or both storage units of fluid is delivered to the vial. This pump is the same as the coil pump used in the fluidic router, except with a longer coil of tubing. The coil holder was developed for this instrument and is now a standard component (Fig 3A; AC1). The vial holder was also developed for this system, and is another example of how an existing component (in this case, a microcentrifuge vial) can be integrated into the MEC system.

To fill Vial 1 in Fig 5B, the titration instrument combined nine unit volumes of water, zero unit volumes of sodium hydroxide solution, and one unit volume of phenolphthalein solution (a mixing ratio of 9:0:1; final NaOH mass concentration = 0). Vial 2 received unit volumes in a ratio of 8:1:1 (NaOH mass concentration = 0.5 × 10^−5^), Vial 3 received a ratio of 7:2:1 (NaOH mass concentration = 1.0 × 10^−5^), and Vial 4 received a ratio of 1:8:1 (NaOH mass concentration = 4.0 × 10^−5^). Each vial received a single unit volume of phenolphthalein solution (a pH indicator that turns from clear to pink at pH > 8.2). The observed colors of the solutions (ranging from clear in Vial 1 to pink in Vial 4 in Fig 5B) confirm the increasing pH from Vial 1 to Vial 4 and the successful operation of the MEC-based titration instrument.

### MEC-based cell culture instrument

Finally, to demonstrate that the MEC system can be used to create research-grade biological instruments, a MEC-based bioreactor was built. The bioreactor (Fig 5C) contains a square-shaped loop of MECs that contains 46 mL of cell culture media. This loop was constructed from a new family of MEC components that interconnect lengths of 0.5-inch diameter rigid plastic tubing. These interconnect MECs are fabricated to the MEC mechanical standards in both size and interface spacing. This allows them to easily mount to our standard baseboard as shown in Fig 5C. The media is recirculated (and cells are kept in suspension) using a stream of air bubbles, although pump MECs could also be used. The bioreactor uses the optical density MEC (OD1 in Fig 3B) to measure the optical density of the cell culture in the media loop.

After sterilizing the MEC bioreactor with a 70% ethanol solution, the bioreactor was filled with yeast extract peptone dextrose (YEPD) media, then seeded with a small amount of *Saccharomyces cerevisiae* yeast culture. The bioreactor was then placed in an incubator at 30°C for 24 hours. An inexpensive data acquisition card (NI USB-6008, National Instruments, Austin, TX) was used to record the voltage output of the optical density MEC, though an Arduino-based microcontroller MEC (under development) could also be used. Data from the optical density MEC is available in S1 File; this file includes the Python program used to analyze the data.

The output of the optical density MEC is plotted versus time in Fig 5D. The slight decrease in growth rate observed at 13 hours likely represents the diauxic shift, the point at which the glucose in the culture media is depleted and the yeast cells must switch their metabolism from glycolysis to the less-favored aerobic oxidation of ethanol. Additionally, the halt in growth observed after 15 hours indicates the depletion of ethanol in the media and the entry of the yeast cells into stationary phase (G_0_). These metabolic transitions are extremely important in yeast biology and even provide insights into human conditions like cancer and aging [10]. The fact that our prototype MEC-based bioreactor is capable of observing these biologically and clinically meaningful metabolic transitions confirms that research-grade instruments can be built using the MEC system.

## Discussion

In this work we presented a roadmap for developing a set of components useful in building biological and chemical instruments. Central to this roadmap are characteristics—packaging, symbolic design, interdisciplinary components, and standardized 3D interfaces—that we feel enable a set of components to adapt to a wide range of applications.

We then used our roadmap to guide the creation of a prototype set of building blocks we call Multifluidic Evolutionary Components. Because the MEC system controls fluid on multiple scales (from nanoliters to milliliters) and integrates multiple fields (fluidics, electronics, mechanics, and optics), MEC blocks can be used to build instruments for many different fields, and we demonstrated this by using MECs to create functional chemical and biological instruments. In building these instruments with our standard MEC components, we demonstrated the utility of the component set and also showed how this component set naturally evolves.

A system of evolutionary components would allow users from a wide range of backgrounds to develop instruments faster and more efficiently. For example, users with limited technical experience and no access to fabrication resources can combine off-the-shelf macroMECs into a working custom instrument. For applications that require the manipulation of very small fluid volumes, the integrated components of microMECs can be used to perform standard microfluidic functions. Users with access to microfabrication resources can combine parts from the library of microMECs into custom microfluidic integrated components that interface easily with the rest of the MEC system. By packaging microfluidic chips in MEC shells, chips from different researchers can be easily connected together to support new applications, thereby encouraging collaboration in the microfluidics community. Finally, as the library of MECs grows and becomes more widely available, industrial users can use MECs to accelerate the development and production of new instruments for the chemical and life sciences.

Our own experience with undergraduate researchers suggests that even individuals with no prior research experience can quickly learn about the MEC system, use MECs to build functional instruments, and even design and fabricate new MECs. Thus far over 50 undergraduates from the University of California, Riverside have worked with the MEC system, and these undergraduates have been able to use MECs to build functional instruments that would not have been practical to build using conventional tools.

Evolutionary components could also prove valuable in resource-limited settings. While first-world hospitals and research universities commonly have rooms full of instruments (each of which typically performs only one or a few different tasks), hospitals and universities in developing parts of the world often do not have these resources. The MEC system could provide researchers and clinicians in resource-limited settings with the “building blocks” they need to construct their own instruments that are custom tailored to their own unique research or diagnostic needs. Having a library of MECs would give these individuals access to a virtually limitless variety of custom instruments. Realizing this vision will require the expansion of the MEC library and the mass-production of MEC parts at low per-unit costs using techniques like injection molding.

A system of standardized components like the MEC system could also help fix the “irreproducible research crisis” facing science today. Recent studies indicate that a significant fraction of published research contains results that can not be reproduced by others [11]. One practical barrier to reproducing another researcher’s results is simply not having access to the equipment needed to reproduce the results. Another barrier to confirming research is the lack of adequate published experimental details about the tools used to perform an experiment. These and other barriers to reproducible research could be eliminated by using standardized components like the MEC system. If a researcher builds their experimental tools using MECs and publishes the schematic, anyone with access to a library of MECs could replicate the researcher’s tools and confirm their results. A set of standardized components for research could have a significant positive impact on the reproducibility of scientific research.

Finally, in addition to research applications, evolutionary components can also be used in a variety of educational applications. For example, the MEC fluidic router in Fig 5A can demonstrate the different types of flow (laminar vs. turbulent) at different flow rates and scales, the MEC titration system shown in Fig 5B could be used to study acid-base equilibrium in a chemistry lab course, and the MEC bioreactor in Fig 5C illustrates concepts of cell growth and metabolism. The National Research Council of the National Academies recently recommended that students from kindergarten through high school supplement their science curriculum by (1) defining and delimiting engineering problems, (2) developing possible solutions, and (3) optimizing the design solution [12]. These engineering activities are a key part of the Next Generation Science Standards that eighteen states are currently adopting. The MEC system supports all three of these engineering activities and can help teachers incorporate engineering practices throughout the science curriculum.

## Supporting Information

S1 File Data from Optical Density MEC during yeast growth curve measurement. ZIP file containing the raw data from the growth curve plot in Fig 5D, obtained from the Optical Density Multifluidic Evolutionary Component (MEC) in the bioreactor instrument shown in Fig 5C. The file also contains the Python program used to convert the raw data into the growth curve plot shown in Fig 5D.
(ZIP)
